# Birdsongs recognition based on ensemble ELM with multi-strategy differential evolution

**DOI:** 10.1038/s41598-022-13957-w

**Published:** 2022-06-13

**Authors:** Shanshan Xie, Yan Zhang, Danjv Lv, Haifeng Xu, Jiang Liu, Yue Yin

**Affiliations:** 1 College of Big Data and Intelligent Engineering, Southwest Forestry University, Kunming, 650000 China; 2 College of Mathematics and Physics, Southwest Forestry University, Kunming, 650000 China; 3 School of Information Science and Technology, Beijing Forestry University, Beijing, 100091 China

**Keywords:** Machine learning, Information technology

## Abstract

Birds are a kind of environmental indicator organism, which can reflect the changes in the ecological environment and biodiversity, and recognition of birdsongs can further help understand and protect birds and natural environment. Extreme learning machine (ELM) has the advantages of fast learning speed and good generalization ability, which is widely used in classification and recognition problems. Input layer weights and hidden layer thresholds are two key factors affecting ELM performance. As one of swarm intelligence optimization methods, differential evolution (DE) can be used to optimize the parameters of ELM. In order to enhance the diversity, convergence speed and global search ability of the DE population, and improve the accuracy and stability of the classification model, this paper proposes a multi-strategy differential evolution method (M-SDE) to optimize the parameters of the ELM. And the differential MFCC feature parameters, extracted from birdsongs, are applied to build classification models of M-SDE_ELM and an ensemble M-SDE_EnELM with optimized ELM for bird species recognition. In the experiments, the ELM models optimized by the swarm intelligence algorithms PSO and GOA are compared and analyzed by hypothesis tests with the M-SDE_ELM and M-SDE_EnELM. Results show that the M-SDE_ELM and M-SDE_EnELM can achieve a classification accuracy of 86.70% and 89.05% in the classification of nine species of birds respectively, and the recognition effect and stability of the M-SDE_EnELM model outperform other models.

## Introduction

Birds play an important role in nature. Knowing the birds in a specific area can help us understand the ecology of the area, and can effectively evaluate the environmental quality of the area's ecology, which is of great significance to the protection of the natural environment. Bird recognition helps us get along better with nature, and also provides a new perspective for researchers to maintain ecological balance and monitor ecology. As the language of birds, birdsongs is an important physiological feature of birds, and there are great differences in the birdsongs of different species of birds^1,2^. Therefore, birds recognition focuses on birdsongs. At present, many researchers have collected birdsongs signals and carried out a lot of research work. With adopting various feature parameters extraction techniques for birdsongs signals, machine learning algorithms are used to classify and recognize birdsongs. Extracting of the exact feature parameters and exploiting better learning algorithm will play a key role in the classification results.

Feature parameters commonly used in birdsongs recognition technology include MFCC, short-time energy (STE), linear predictive cepstral coding (LPCC) and linear predictive coding (LPC), etc. For example, Wang et al. took eight kinds of birds as the research object, and divided the birdsongs into bird tweet and bird sing to extract their MFCC feature parameters, and used the dual gaussian mixture model for training and recognition^3^. Xu et al. studied birdsongs recognition based on syllable length, MFCC, and DTW model based on LPC, combined with time–frequency texture feature and multi-label classifiers. With eleven kinds of birds as the research object, it selected the optimal feature parameters and classifiers to improve the recognition effect of a single classifier^4^.

Classical classifiers include random forest, decision tree, gaussian mixture model, neural network, ELM, etc. ELM is a randomized fast learning algorithm with good generalization ability^5,6^. Using ELM to study classification and recognition problems has become a research hotspot. For example, Xue et al. classify and recognize power quality events based on wavelet transform and ELM, which can effectively recognize eight kinds of disturbances and have strong robustness^7^. Lin et al. assisted in the diagnosis of Alzheimer's disease based on ELM, and the accuracy of this method in diagnosing Alzheimer's disease reached 87.62%^8^. Venkatalakshmi et al. extracted breast X-ray image feature set and combined ELM classifier to classify normal, malignant and benign breast cancer. The accuracy, sensitivity and specificity of the method are better than that of similar technology^9^. Kashif et al. proposed an ELM-based consonant phoneme recognition model for the accent recognition of different pronunciations of English consonant phonemes by native Arabic speakers, the accuracy of the model reached 88%^10^.

Because ELM generally randomly generates input layer weights and hidden layer thresholds, and then obtains output weights through calculations. There is no uniform form for the selection of parameters, and only a large amount of training and learning can be used to obtain the optimal parameter value. This method takes a long time because of calculation complexity. The final result may not be the optimal solution, and the performance of the classifier is unstable^11^. Therefore, it is necessary to use intelligent algorithm to optimize ELM parameters to make the classifier achieve better results. DE is a population-based random search algorithm^12,13^, which conducts intelligent search through mutation and crossover, and ensures that the best individual can be further utilized. With fast convergence speed and good global search performance, DE is one of the most powerful and universal evolutionary optimizers in continuous parameter space. For example, to reduce the prediction time of ELM and avoid falling into local optimality, Yang et al. proposed a differential evolution coral reef optimization algorithm with hybrid DE and metaheuristic coral reef optimization to balance exploration and development capabilities to achieve better performance^14^. Dahou et al. combined DE and convolutional neural networks (CNN) to solve the problem of Arabic sentiment classification, and used DE algorithm to optimize CNN parameters. Experiments show that DE-CNN has good performance in terms of accuracy and time consumption^15^. Li et al. used principal component analysis to reduce the dimensionality of the input feature and used the sequence floating backward algorithm to perform feature selection, and then input the optimal feature set into the differential evolution ELM to evaluate the transient stability of the power system. Compared with other ELMs, this model greatly improved its performance in transient stability classification evaluation^16^.

However, the standard DE algorithm often leads to premature convergence and search stagnation^17^. Therefore, many scholars conducted research on DE algorithm improvement. For example, Singh et al. used multi-objective DE to adjust the initial parameters of CNN and the optimized CNN can effectively classify chest CT images for COVID-19^18^. A memetic differential evolution algorithm was proposed to solve the problem of text clustering, improved the mutation strategy of DE and mixed it with the memetic algorithm, and was superior to other clustering algorithms based on AUC measurement, F-measure, statistical analysis and existing text clustering algorithms^19^. Vivekanandan et al. used “DE/rand/2/exp” as the differential strategy to select optimal feature of cardiovascular disease, using fuzzy analytic hierarchy process and feedforward neural network to predict heart disease, the accuracy of the model reached 83%^20^. Duan et al. combined “DE/best/2” mutation operator and “DE/rand/2” mutation operator to form a dual-strategy and dynamically adjusted control factor $\uplambda$ during the evolution process, this algorithm can significantly improve the global optimization performance^21^. However, the mutation strategies adopted by these articles for DE are single strategy or dual strategy, and they all used a single classifier for classification. In order to improve population diversity, convergence speed and global search ability, this paper proposes a multi-strategy mutation of DE algorithm. And the classification model of ensemble multiple DE-optimized ELMs will be built to enhance the recognition effect and generalization ability.

This paper takes birdsongs as the research object. Firstly, the MFCC feature parameters of the birdsongs data are extracted, and in order to maintain the time domain continuity of the audio signal^22^, performing differential calculation on MFCC. Secondly, a multi-strategy mutation is formed by combination of three strategies, while using adaptive adjustment control parameters (scaling factor F and crossover probability CR) to improve the standard DE. The input layer weights and the hidden layer thresholds of ELM are adjusted through the DE. Finally, we ensemble optimized ELM model to classify birdsongs. This model can better solve the problems of unstable performance of ELM classifier and difficulty in determining the number of optimal hidden layer neurons in birdsongs recognition.

The main contributions of this paper can be summarized as follows:

1. Adopt the multi-strategy mutation in DE algorithm (M-SDE) to improve the population diversity and global search ability;
2. Use the M-SDE algorithm to optimize the hidden layer thresholds and input layer weights of the ELM model;
3. Extract the differential MFCC feature parameters of birdsongs, and build the ensemble optimized ELM (M-SDE_EnELM) to improve model stability and recognition accuracy for birdsongs.

The rest of this paper is organized as follows: Firstly, the ELM and differential evolution are described. Secondly, we propose multi-strategy differential evolution algorithm. Thirdly, we introduce the MFCC feature parameters extraction process of birdsongs and the birdsongs recognition model based on ensemble ELM with multi-strategy differential evolution algorithm. Fourthly, experimental results and limitations are discussed. Finally, we give the conclusions.

## Extreme learning machine

**Figure 1:**
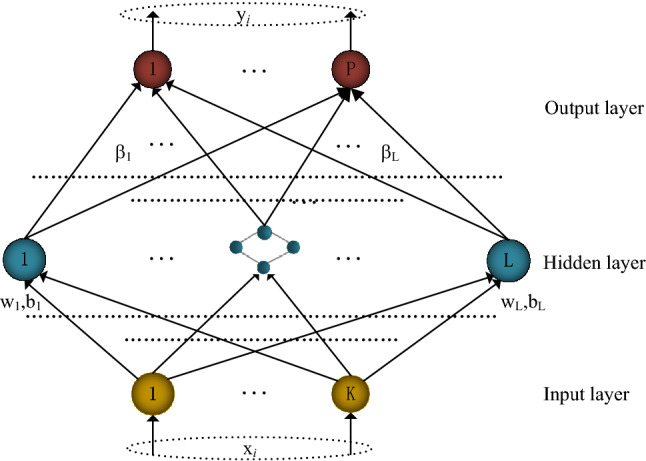
Topological diagram of SLFNs.

ELM is an algorithm for training single layer feedforward neuron networks (SLFNs)^23^, which can effectively reduce the model runs time and produce good generalization performance. The topology of SLFNs is shown in Fig. 1.

SLFNs is composed of input layer, hidden layer and output layer. Assuming that the input layer has $K$ nodes. $K$ describes the number of features; the hidden layer has $L$ nodes, and $L$ describes the number of neurons in the hidden layer; the output layer has $P$ nodes, $P$ is the number of sample categories. SLFNs can be represented by triples $(K,L,P)$. Suppose training set:

1 $$\begin{array}{rcl}\mathrm \ Train={\{{(x_i,y_i)}\mid\begin{array}{ccc}x_i={\lbrack x_{i1},x_{i2},\dots,x_{iK}\rbrack}^T\in R^K,\\y_i={\lbrack y_{i1},y_{i2},\dots,y_{iP}\rbrack}^T\in R^P\end{array}\}},1\leq i\leq N\end{array}$$

Where $N$ is the number of training samples, ${{x}_{i}=[{x}_{i1},{x}_{i2},\dots ,{x}_{iK}]}^{T}\in {R}^{K}$ is the feature of the sample, ${{y}_{i}=[{y}_{i1},{y}_{i2},\dots ,{y}_{iP}]}^{T}\in {R}^{P}$ is the category to which the sample $i$ belongs to $P$ categories and ${y}_{ip}\in \left\{\mathrm{0,1}\right\},p=1,\dots ,P$. Then the SLFNs with $L$ hidden nodes can be expressed as

2 $$f\left({x}_{i}\right)=h\left(x\right)\beta =\sum_{j=1}^{L}{\beta }_{j}g\left({w}_{j}\cdot {x}_{i}+{b}_{j}\right),i=\mathrm{1,2},\dots ,N$$

where $h\left(x\right)$ is the feature mapping function, $x$ is the input of the neural network, ${{w}_{j}=({w}_{j1},{w}_{j2},\dots ,{w}_{jK})}^{T}$ is the input weight, that is, the weight vector to connecting the input layer node and the *j*th node in the hidden layer, ${{\beta }_{j}=({\beta }_{j1},{\beta }_{j2},\dots ,{\beta }_{jK})}^{T}$ is the output weight, that is, the weight vector connecting the *j*th node of the hidden layer and the output layer node, ${b}_{j}$ is the threshold of the hidden layer, that is, the bias of the *j*th unit of the hidden layer. In ELM, the input weights and hidden layer thresholds are randomly initialized and generated, and the corresponding output weights are obtained. The goal of SLFNs learning is to minimize the output error, that is, ${\beta }_{j}$ satisfies Eq. (3)

3 $$f\left({x}_{i}\right)=\sum_{j=1}^{L}{\beta }_{j}g\left({w}_{j}\cdot {x}_{i}+{b}_{j}\right)={y}_{i},i=\mathrm{1,2},\dots ,N$$

It can be expressed as a matrix

4 $$H\beta =Y$$

5 $$H\left({w}_{1},\dots ,{w}_{L},{b}_{1},\dots ,{b}_{L},{x}_{1},\dots ,{x}_{L}\right)=\left(\genfrac{}{}{0pt}{}{\begin{array}{c}g\left({w}_{1}\cdot {x}_{1}+{b}_{1}\right) \dots g\left({w}_{L}\cdot {x}_{1}+{b}_{L}\right)\\ \dots \dots \dots \end{array}}{g\left({w}_{1}\cdot {x}_{N}+{b}_{1}\right) \dots g\left({w}_{L}\cdot {x}_{N}+{b}_{L}\right)}\right)$$

6 $${\beta =({{\beta }_{1}}^{T},{{\beta }_{2}}^{T},\dots ,{{\beta }_{L}}^{T})}^{T}$$

7 $${Y=({{y}_{1}}^{T},{{y}_{2}}^{T},\dots ,{{y}_{N}}^{T})}^{T}$$

where $H$ is the output matrix of the hidden layer, $\beta$ is the output weight, and $Y$ is the expected output. ELM, a simple learning method for SLFNs, needs to search the optimal parameters ${\widehat{w}}_{j},{\widehat{b}}_{j},{\widehat{\beta }}_{j}$ to satisfy Eq. (8):

8 $$\Vert H\left({\widehat{w}}_{j},{\widehat{b}}_{j}\right){\widehat{\beta }}_{j}-Y\Vert= \underset{w,b,\beta }{\mathrm{min}}\Vert H\left({w}_{j},{b}_{j}\right){\beta }_{j}-Y\Vert$$

The least square solution of Eq. (8) can be expressed as

9 $$\widehat{\beta }={H}^{+}Y$$

where ${H}^{+}$ is the Moore–Penrose pseudo-inverse matrix of matrix $H$.
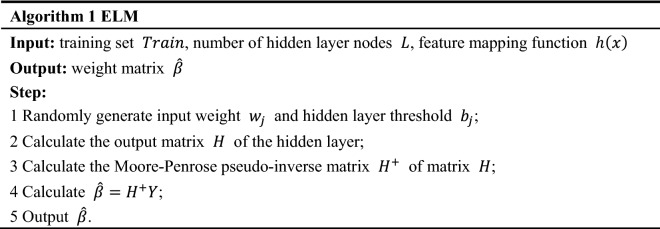


## Differential evolution

Differential evolution is a swarm intelligent optimization method. It mainly works in four steps, initializing the population generation, mutation operation, crossover operation and selection operation. However, the individual in the population may cause the value of the individual to exceed the given maximum and minimum range after mutation and crossover, so boundary condition processing operation must be performed after the crossover operation.

1. Initialize the generated population
  The individuals in the population can be expressed as: (the population size is $NP$, the number of iterations is $T$, and each individual is composed of a $D$-dimensional vector.)
  10 $${x}_{i}\left(j\right)=\left({x}_{i,1}\left(j\right),{x}_{i,2}\left(j\right),{x}_{i,3}\left(j\right),\dots ,{x}_{i,D}\left(j\right)\right),1\le i\le NP,1\le j\le T$$
  The initial population individuals are generally randomly generated within a given constraint boundary, as follows:
  $${x}_{i,d}\left(j\right)=Xmin+rand\left(\mathrm{0,1}\right)\left(Xmax-Xmin\right),$$
  11 $$1\le i\le NP,1\le j\le T,1\le d\le D$$
  $Xmax$ and $Xmin$ represent the upper and lower bounds of the individual values of the population respectively, and rand (0,1) refers the generation of a uniformly distributed random number between (0,1).
2. Mutation operation
  The mutation vector is generated by the individual of its parent population through the mutation strategy. The mutation strategy is represented by “DE/x/y” and where x represents the vector such as random vector, best vector and current vector; y is the number of difference vectors. The commonly used mutation strategies are shown in Table 1.

**Table 1:** Mutation strategy.

Mutation strategy	Formula
DE/rand/1	${v}_{i}\left(j\right)= {x}_{r1}\left(j\right)+F({x}_{r2}\left(j\right)-{x}_{r3}\left(j\right))$
DE/best/1	${v}_{i}\left(j\right)= {x}_{best}\left(j\right)+F({x}_{r1}\left(j\right)-{x}_{r2}\left(j\right))$
DE/current to best/1	${v}_{i}\left(j\right)= {x}_{i}\left(j\right)+F\left({x}_{best}\left(j\right)-{x}_{i}\left(j\right)\right)+F\left({x}_{\mathrm{r}1}\left(j\right)-{x}_{\mathrm{r}2}\left(j\right)\right)$
DE/best/2	${v}_{i}\left(j\right)= {x}_{best}\left(j\right)+F\left({x}_{r1}\left(j\right)-{x}_{r2}\left(j\right)\right)+F\left({x}_{r3}\left(j\right)-{x}_{r4}\left(j\right)\right)$
DE/rand/2	${v}_{i}\left(j\right)= {x}_{\mathrm{r}1}\left(j\right)+F\left({x}_{r2}\left(j\right)-{x}_{r3}\left(j\right)\right)+F\left({x}_{r4}\left(j\right)-{x}_{r5}\left(j\right)\right)$
  Where *F* is the scaling factor and 0 $\le$ *F* $\le$ 2; $r1,$ $r2, r3, r4$ and $r5$ are randomly generated in the parent population and $r1\ne r2\ne r3\ne r4\ne r5$; ${x}_{\mathrm{best}}$ is the best individual with the best fitness in the parent population; ${x}_{i}$ is the current corresponding parent population individual.
3. Crossover operation
  12 $${u}_{i,d}(j)=\left\{\begin{array}{c}{v}_{i,d}\left(j\right), if \, rand\left(\mathrm{0,1}\right)<CR \, or \, randi\left(1,d\right)=j\\ {x}_{i,d}\left(j\right), if \, rand(\mathrm{0,1})>CR \, and \, randi\left(1,d\right)\ne j\end{array}\right.$$
  where $CR$ is the crossover probability and $0\le CR\le 1$.
4. Boundary condition processing operation
  13 $${u}_{i,d}(j)=\left\{\begin{array}{l}\left(Xmax-Xmin\right)*rand\left(\mathrm{0,1}\right)+Xmin, \, if \, {u}_{i,j}<Xmin \, or \, {u}_{i,j}>Xmax\\ {u}_{i,d}\left(j\right) , \, else\end{array}\right.$$
5. Selection operation
  14 $${x}_{i}(j+1)=\left\{\begin{array}{l}{u}_{i}\left(j\right), \, if \, f\left({u}_{i}\left(j\right)\right)<f({x}_{i}\left(j\right))\\ {x}_{i}\left(j\right), \, else\end{array}\right.$$
  With greedy criterion, the DE algorithm selects individuals from $u$ as the individuals of the next generation population $x$, $f\left({u}_{i}\left(j\right)\right)$ represents the fitness of the *j*th generation individual in the crossover population $u$, and $f\left({x}_{i}\left(j\right)\right)$ represents the fitness of the *j*th generation individual in the parent population $x$.

## Multi-strategy differential evolution

Differential evolution performs intelligent search through mutation and crossover, and then selects the optimal individual. It has fast convergence speed and good global search performance. So, it can be used to optimize the parameters of the classifier. Because the input layer weights and hidden layer thresholds of ELM are generated randomly, the classifier becomes unstable due to the randomness of input layer weights and hidden layer thresholds. The method of running step by step to find the optimal input layer weights and hidden layer thresholds takes a long time, and the result may not be the optimal solution. Therefore, this paper uses differential evolution algorithm to optimize ELM parameters and improve the stability of the classifier, to make the classifier achieve better results.

In this study, we propose a multi-strategy differential evolution (M-SDE) to optimize parameters of ELM. Combination of three strategies forms a multi-strategy mutation. Three strategies are as follows.

1. “DE/rand/2” with strong global search performance and maintain population diversity;
2. “DE/best/2” with strong local development ability and fast convergence speed;
3. “DE/current to best/1” with the ability to maintain the diversity of the population and high convergence precision.

**Figure 2:**
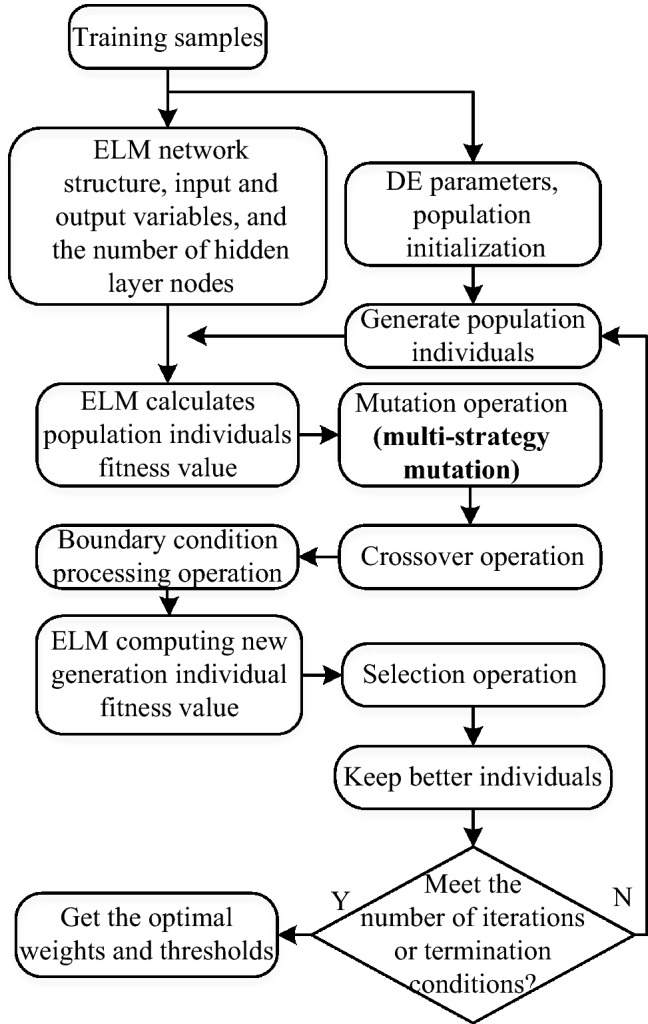
Optimized parameters of ELM with M-SDE.

While using adaptive adjustment control parameters (scaling factor F and crossover probability CR) to improve the standard DE, Fig. 2 shows the process of DE optimizing ELM.
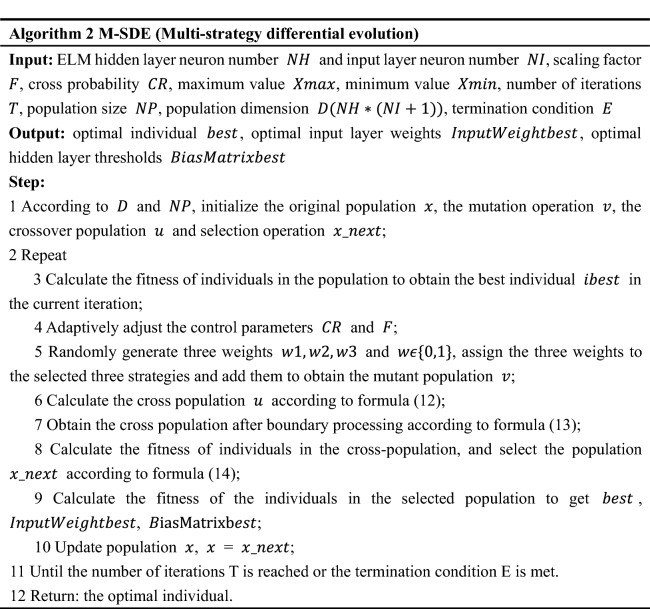

### Ensemble ELM with multi-strategy differential evolution

ELM classifier has a certain instability, so it is often necessary to improve the stability and accuracy of the classifier through ensemble methods. Therefore, this paper takes the value range of hidden layer neurons of ELM from four to ten times of the number of sample features, uses the majority voting algorithm to ensemble ten ELM models, and then uses the ensemble model to classify the test data.

This paper takes birds as the research object, collects birdsongs data, develops the study of birdsongs recognition based on M-SDE optimization ensemble ELM, and extracts the MFCC feature of birdsongs, establishes a birdsongs recognition model based on M-SDE optimized ensemble ELM.

### MFCC feature extraction of birdsongs

**Figure 3:**
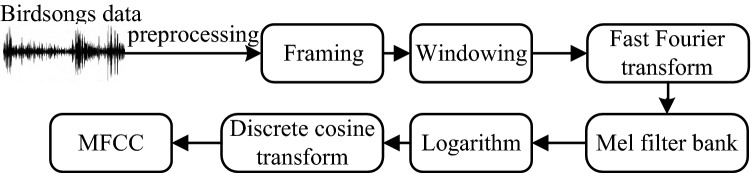
Process of extracting MFCC feature parameters.

The MFCC is proposed on the basis of auditory feature of the human ear that fully simulates the auditory feature of the human ear. It combines the human auditory feature and sound production, and is a sound feature parameter widely used at present^24–26^. Firstly, the input sound signal is performed denoising processing. Secondly, through endpoint detection, the effective voice segment is determined. Finally, the differential MFCC feature parameters of birdsongs signals are extracted to obtain its feature matrix. The process of extracting MFCC feature parameters is shown in Fig. 3.

In this paper, the 13-dimensional MFCC feature parameters are extracted. In order to maintain the time domain continuity of the audio signal, the 13-dimensional ΔMFCC feature parameters and the 13-dimensional ΔΔMFCC feature parameters are obtained through the first-order difference and the second-order difference, respectively, and the three are fused (MFCC + ΔMFCC + ΔΔMFCC) to obtain the 39-dimensional differential MFCC feature parameters.

The differential formula is as follows:

15 $${d}_{t}=\left\{\begin{array}{l}{C}_{t+1}-{C}_{t},\quad t<K\\ \frac{\sum_{k=1}^{K}k({C}_{t+k}-{C}_{t-k})}{\sqrt{2\sum_{k=1}^{K}{k}^{2}}},\quad other\\ {C}_{t}-{C}_{t-1},\quad t\ge Q-K\end{array}\right.$$

Among them, ${C}_{t}$ is the *t*th feature parameter, ${C}_{t-1}$ is the *t* − 1th feature parameter, ${C}_{t+1}$ is the *t* + 1th feature parameter, $Q$ is the dimension of the feature parameter, ${d}_{t}$ is the *t*th first order difference, and $K$ is the time difference of the first order derivative is usually 1 or 2. In this paper, we take $K$=2. Substituting the first order difference calculated for the first time into Eq. (15) again can get the second order difference.

### A birdsongs recognition model based on ensemble ELM with M-SDE

The birdsongs recognition model based on optimized ELM mainly includes four modules, namely, the establishment of training set and test set, training of M-SDE_ELM model, establishment of ensemble optimized ELM (M-SDE_EnELM) and prediction, details as follows:

1. Divide the differential MFCC feature data of birdsongs into training set and test set at a ratio of 7:3;
2. Generate optimized ELM (M-SDE_ELM) by optimizing the input layer weights and hidden layer thresholds of ELM through DE, where the mutation strategy of each iteration of DE is randomly generated by the three selected strategies (“DE/best/2”, “DE/rand/2”, “DE/current to best/1”);
3. Ensemble n (n = 10) M-SDE_ELM to form the M-SDE_EnELM ensemble model;
4. The majority voting algorithm are used in the trained M-SDE_EnELM ensemble model to classify and recognize birds.

**Figure 4:**
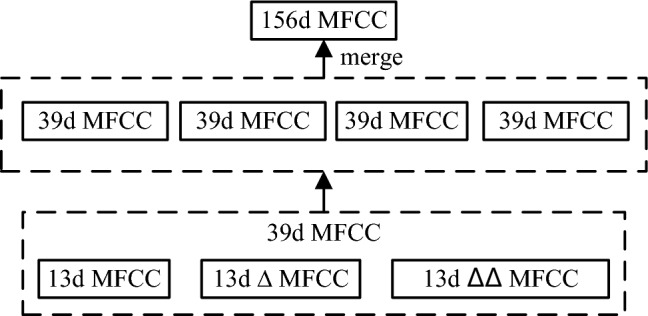
Extraction of birdsongs feature parameters.

**Figure 5:**
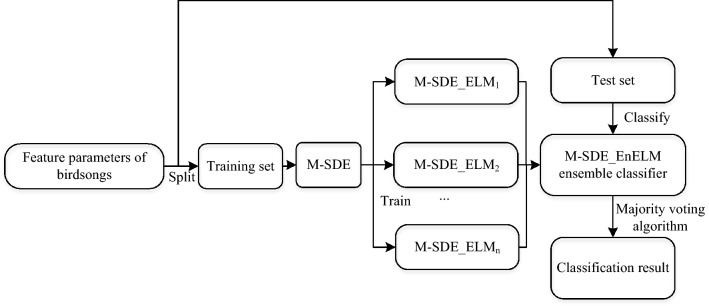
Process of M-SDE_EnELM.

Figures 4 and 5 show the process of constructing a birdsongs recognition model based on ensemble ELM with M-SDE. Figure 4 shows the extraction of birdsongs feature parameters. Figure 5 shows the M-SDE_EnELM model.

The majority voting algorithm is as follows.

16 $$H(x)=\underset{1\le j\le P}{\mathit{argmax}}\sum_{i=1}^{n}{y}_{ij}(x)$$

where $n$ is the number of ensemble model base classifiers, in this paper $n$=10. If the *i-th* base classifier classifies the sample $x$ as class $j$, then ${y}_{ij}(x)$=1, otherwise ${y}_{ij}\left(x\right)$=0.

## Experiments and analysis

### Data preprocessing

The collection of birdsongs data is mainly collected by the internet of things and crawler, supplemented by manual collection. Manual collection is to collect birdsongs through mobile phone or voice recorder; the internet of things collection is to collect birdsongs by designing the internet of things collection module; crawler collection is to crawl bird audios from the bird audios website^27^, the experimental data in this paper is obtained through web crawler.

This paper collected nine kinds of bird audios, namely Short-eared Owl, Whimbrel, Cormorant, Long-eared Owl, Sparrowhawk, Common Quail, Common Crane, Goshawk and Kestrel. The experimental bird audios are preprocessed and the unified data format is .wav format. The sound channel is mono, and the frequency is 16000HZ. After MFCC feature extraction, the feature matrix of birdsongs is obtained. The feature data is divided into training set and test set according to the ratio of 7:3, and four frames of audios data (each frame is 39d MFCC) are combined into one frame as a sample (156d MFCC). The training samples and test samples of each bird are shown in Table 2.

**Table 2:** Datasets of birdsongs samples.

Category of bird	Number of audios	Training set	Test set
Short-eared owl	11	880	377
Cormorant	9	427	183
Whimbrel	21	588	252
Long-eared owl	34	567	243
Sparrowhawk	11	593	254
Common Crane	14	2164	927
Kestrel	10	2031	870
Goshawk	11	1420	608
Common Quail	10	2516	1078

### Experimental schemes

To verify the effectiveness of the proposed method, two groups of experiments are designed in this paper, and each group of experiment is run 10 times respectively, and the average result is taken as the final result. At the same time, in order to compare the difference and stability of the proposed method with other methods, hypothesis tests (t-test and F-test) analysis were performed on the 10 times run results of the two groups of experiments.

**Figure 6:**
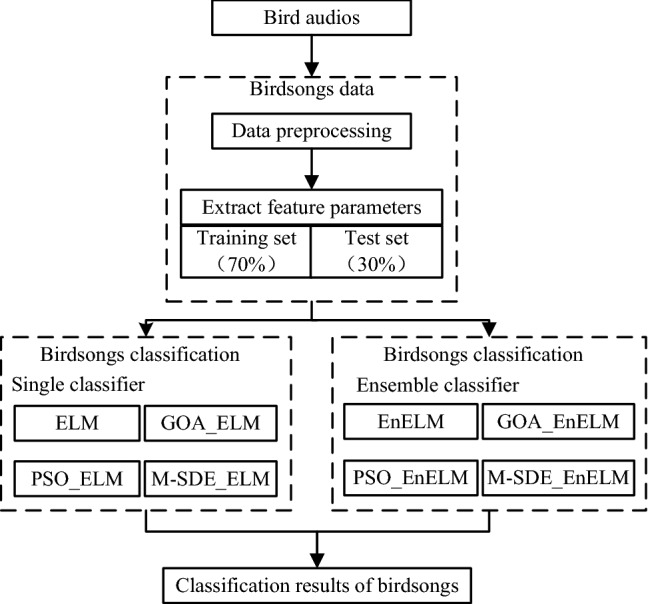
Experimental design process.

The first set of experiment is the single classifier comparison experiment. That is, original ELM model compared with the optimized ELM model by the heuristic algorithm. Grasshopper optimization algorithm (GOA)^28^ and particle swarm optimization algorithm (PSO)^29^ are population-based intelligent optimization algorithms which are widely used and have good results. To better compare the recognition effect of the M-SDE_ELM model, the GOA_ELM and PSO_ELM models (ELM is optimized by PSO and GOA, respectively.) are compared with it. The second set of experiment is the ensemble classifier comparison experiment. The ensemble original ELM model compares ensemble optimized ELM. The experimental process is shown in Fig. 6.

The parameters of the groups are as follows: the number of hidden layer neurons: n; population size: NP; max iteration: T; the feature dimension of the sample: df; the number of base classifiers: nc; linearly decrease parameter limit: c; velocity upper limit: vmax; target error: minerr; inertia weight limit: w; learning factor: C; cross probability: CR; fitness threshold: y; scaling factor: F0, F.

The experimental parameter settings of two groups are shown in Table 3.

**Table 3:** Setting of experimental parameters.

Model		Parameter settings
Single classifier	ELM	n = [df*4, df*10]
	GOA_ELM	n = [df*4, df*10], c = [0.00004, 1], NP = 100, T = 30
	PSO_ELM	n = [df*4, df*10], NP = 100, vmax = 2, minerr = 0.00001, w = [0.3, 0.9], C = 2, T = 30
	M-SDE_ELM	n = [df*4, df*10], NP = 100, CR = 0.5*(1 + rand), y = 10^−6, F0 = 0.4, F = F0*2.^exp(1 − T/(T + 1 − t)), T = 30
Ensemble classifier	EnELM	n = [df*4, df*10], nc = 10
	GOA_EnELM	n = [df*4, df*10], NP = 100, nc = 10, c = [0.00004, 1], T = 30
	PSO_EnELM	n = [df*4, df*10], NP = 100, nc = 10, vmax = 2, minerr = 0.00001, w = [0.3, 0.9], C = 2, T = 30
	M-SDE_EnELM	n = [df*4, df*10], NP = 100, nc = 10, CR = 0.5*(1 + rand), y = 10^−6, F0 = 0.4, F = F0*2.^exp(1 − T/(T + 1 − t)), T = 30

In the second experimental scheme, ELM, GOA_ELM, PSO_ELM and M-SDE_ELM are used as the base classifiers of the four ensemble classifiers.

### Analysis of experimental results

The experiment process in this paper is based on Matlab R2018b. After training the model in the experiment scheme with the training set, test it with the test set to analyze the recognition effect. Accuracy, F1_score and precision are calculated based on the confusion matrix, which are used as indicators to evaluate the classification model.

1. Single classifier
  The classification performance of the four models established, including ELM, GOA_ELM, PSO_ELM, and M-SDE_ELM, on the test set are shown in Table 4.

**Table 4:** Performance of single classifier.

Model	Accuracy (mean ± std)	F1_score(mean ± std)	Precision (mean ± std)
ELM	85.40 ± 0.66%	0.8470 ± 0.0064	0.8560 ± 0.0057
GOA_ELM	85.51 ± 0.56%	0.8468 ± 0.0073	0.8568 ± 0.0077
PSO_ELM	86.00 ± 0.69%	0.8546 ± 0.0080	0.8636 ± 0.0080
M-SDE_ELM	86.70 ± 0.33%	0.8614 ± 0.0041	0.8702 ± 0.0046
  The accuracy of ten runs of each classifier is shown in Fig. 7.

**Figure 7:**
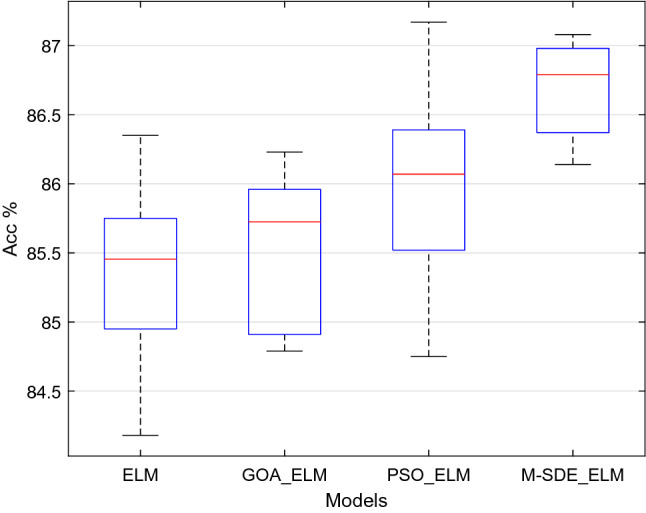
Single classifier experiments.
  In Table 4 and Fig. 7, it shows that on the aspect of accuracy rate, M-SDE_ELM is superior to other three models, and the same as the F1_score and precision rank. On the aspect of standard deviation, the data of M-SDE_ELM is the lowest than other three models which reflects that it has a more stable performance among all the models.
  Hypothesis tests (t-test and F-test) are performed on the 10-times recognition accuracy of M-SDE_ELM with the other three classification models, and the results are shown in Table 5.

**Table 5:** Hypothesis tests of single classifier.

Model	t-test	F-test
M-SDE_ELM + ELM	p = 0.0008	p = 0.0567
M-SDE_ELM + GOA_ELM	p = 6.6160e-05	p = 0.1442
M-SDE_ELM + PSO_ELM	p = 0.0083	p = 0.0424
  It can be seen from Table 5 that the t-tests are performed on M-SDE_ELM with the other three models, the p-values of the test results are all less than 0.05, indicating that the null hypotheses are rejected, that is, the mean of the recognition accuracy of M-SDE_ELM with the other three models have significant difference. F-tests for M-SDE_ELM with ELM and GOA_ELM, p-values are greater than 0.05, indicating acceptance of the null hypotheses, that is, the stability of M-SDE_ELM with ELM and GOA_ELM are consistent. While the p-value of F-test for M-SDE_ELM with PSO_ELM is less than 0.05, indicating that the null hypothesis is rejected, that is, the stability of M-SDE_ELM with PSO_ELM is different, and M-SDE_ELM is relatively stable. From the mean and standard deviation of M-SDE_ELM, the mean of M-SDE_ELM is the largest and the standard deviation is the smallest, so M-SDE_ELM outperforms the other three models.
  It shows that the established M-SDE_ELM classifier has relatively good recognition effect and generalization performance.
  To analyze and compare the efficiency of M-SDE_ELM, PSO_ELM, GOA_ELM three methods for birdsongs classification, the number of iterations and classification accuracy of the three methods are shown in Fig. 8.

**Figure 8:**
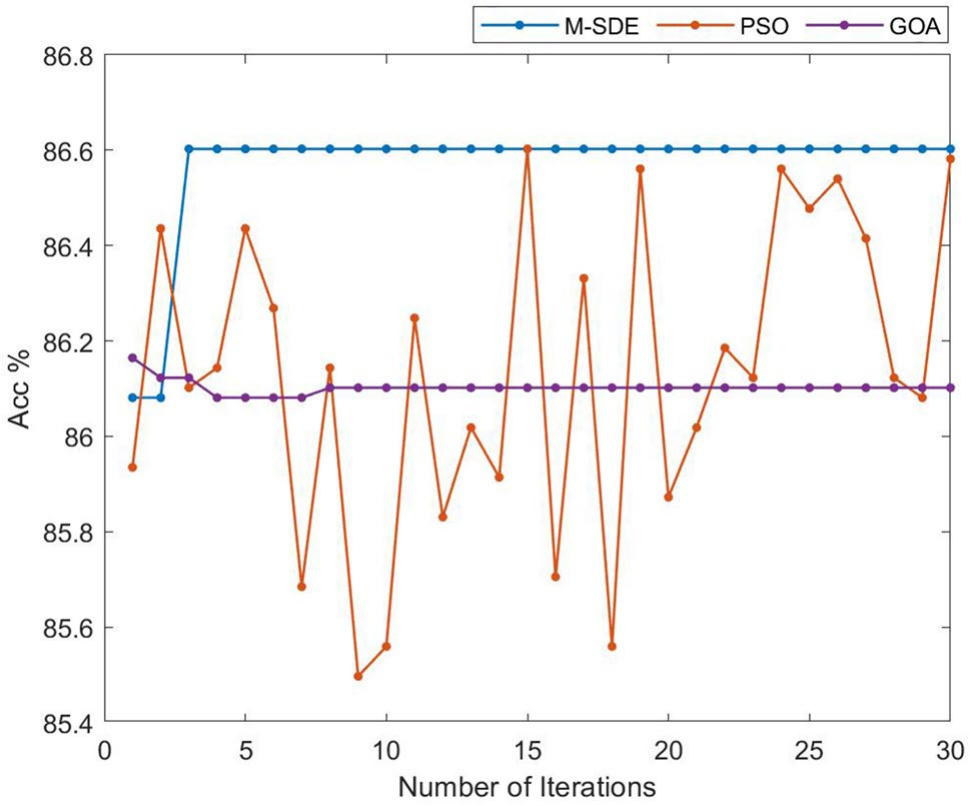
Comparison of iteration and accuracy of three methods.
  It can be seen from Fig. 8 that compared with PSO and GOA, the M-SDE method can converge at the fastest speed and obtain higher classification accuracy, and at the same time, the accuracy fluctuates less and obtains relatively stable performance.
2. Ensemble classifier
  The above four single classification models are used as the base classifiers to construct the ensemble classification models such as EnELM, GOA_EnELM, PSO_EnELM and M-SDE_EnELM, and the classification performance of the test set is shown in Table 6.

**Table 6:** Performance of ensemble classifier.

Model	Accuracy (mean ± std)	F1_score (mean ± std)	Precision (mean ± std)
EnELM	87.88 ± 0.30%	0.8763 ± 0.0036	0.8852 ± 0.0040
GOA_EnELM	87.99 ± 0.21%	0.8758 ± 0.0026	0.8850 ± 0.0024
PSO_EnELM	88.87 ± 0.19%	0.8886 ± 0.0021	0.8978 ± 0.0024
M-SDE_EnELM	89.05 ± 0.19%	0.8887 ± 0.0030	0.8978 ± 0.0029
  The accuracy of ten runs of each classifier is shown in Fig. 9.

**Figure 9:**
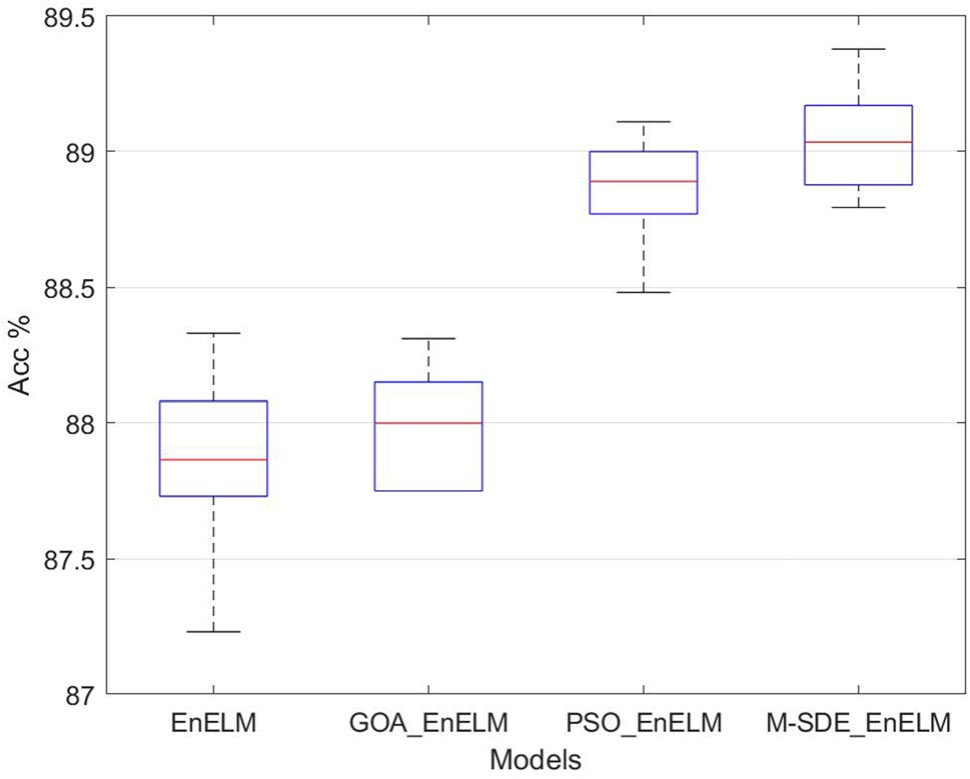
Ensemble classifier experiments.
  From Table 6 the accuracy rate of M-SDE_EnELM is 89.05%, which is 1.17% higher than EnELM, 1.06% higher than GOA_EnELM, and 0.18% higher than PSO_EnELM. From the comparison of Table 4, Table 6 and Fig. 9, it can be seen that performance of M-SDE_EnELM are best among all classification models, and its accuracy is 0.18–3.65% higher than the other seven models. Its F1_score is 0.01–4.19% higher than the other seven models. And it has the same precision as PSO_EnELM and 1.26–4.18% higher than the other six models.
  Hypothesis tests (t-test and F-test) are performed on the 10-time recognition accuracy of M-SDE_EnELM with the other three classification models, and the results are shown in Table 7.

**Table 7:** Hypothesis tests of ensemble classifier.

Model	t-test	F-test
M-SDE_EnELM + EnELM	p = 3.4703E-06	p = 0.1820
M-SDE_EnELM + GOA_EnELM	p = 3.4553E-07	p = 0.7460
M-SDE_EnELM + PSO_EnELM	p = 0.0243	p = 0.9533
  From Table 7, the t-tests are performed on M-SDE_EnELM with the other three models respectively, and the p-values of the test results are all less than 0.05, indicating that the null hypotheses are rejected, that is, the mean of the recognition accuracy of M-SDE_EnELM with the three models have significant difference. F-tests are performed on M-SDE_EnELM with the other three models respectively, and p-values are greater than 0.05, indicating acceptance of the null hypotheses, that is, the stability of M-SDE_EnELM with the three models are consistent. From the mean and standard deviation of M-SDE_EnELM, the mean of M-SDE_EnELM is the largest and the standard deviation is the smallest, so M-SDE_EnELM outperforms the other three models.
  The comparison of the experimental results of all models are shown in Fig. 10. It can be seen that the established M-SDE_EnELM classifier has better recognition effect and generalization performance for the birdsongs dataset. As a whole, the proposed M-SDE_EnELM model has better classification results than other models in the experimental schemes, and achieves better recognition results.

**Figure 10:**
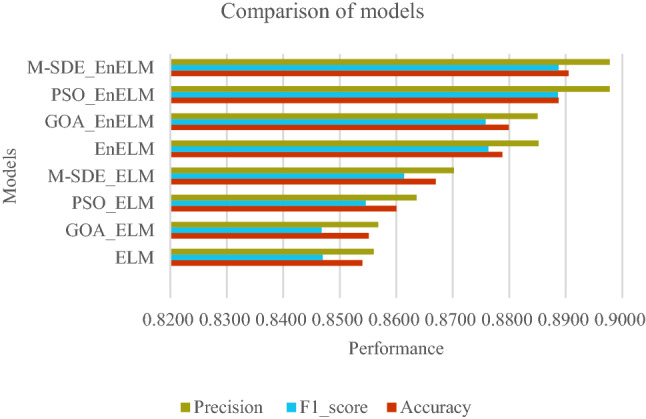
Comparison of models.

### Model ablation

The M-SDE proposed in this paper adopts three different mutation strategies in the mutation stage. In order to further verify the advantages brought by the mixing of the three strategies, two groups of experiments are designed for comparison in this section, namely the single-strategy differential evolution algorithm optimization ELM experiment and the dual-strategy differential evolution algorithm optimization ELM experiment.

1. Single-strategy vs multi-strategy
  The experimental settings of the single-strategy differential evolution algorithm are listed in Table 8. In this group of experiments, except for the different settings of the strategy and the M-SDE, other parameters are consistent with the M-SDE.

**Table 8:** Settings of the single-strategy models.

Model	Mutation strategy
DE_B_ELM	DE/best/2
DE_R_ELM	DE/rand/2
DE_C_ELM	DE/current to best/1
  In the experiment, each model was run independently for ten times, and the performance of each model is shown in Table 9.

**Table 9:** Performance comparison between single-strategy and multi-strategy of models.

Model	Accuracy (mean ± std)	F1_score (mean ± std)	Precision (mean ± std)
DE_B_ELM	85.53 ± 0.46%	0.8502 ± 0.0057	0.8577 ± 0.0063
DE_R_ELM	85.08 ± 0.70%	0.8447 ± 0.0082	0.8513 ± 0.0081
DE_C_ELM	85.18 ± 0.73%	0.8439 ± 0.0099	0.8532 ± 0.0102
M-SDE_ELM	86.70 ± 0.33%	0.8614 ± 0.0041	0.8702 ± 0.0046
  The accuracy of ten runs of each classifier is shown in Fig. 11.

**Figure 11:**
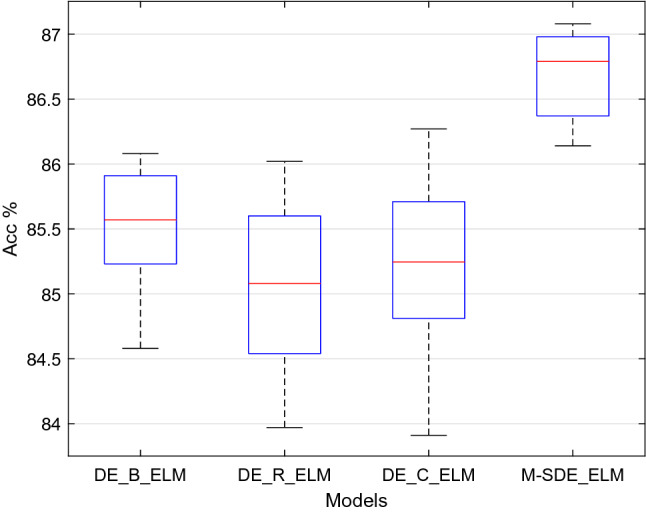
Comparison single-strategy models with multi-strategy models.
  From Table 9, for the M-SDE_ELM model the accuracy is 86.70%, 1.17–1.62% higher than the other three single-strategy models, and its F1_score and precision are 1.12–1.75% and 1.25–1.89% higher than those of the three single-strategy models, respectively. In terms of the standard deviations, M-SDE_ELM has the least fluctuation. Combining with Fig. 11, M-SDE is more stable and has better performance than the three single-strategy models.
  Therefore, we can conclude that M-SDE_ELM is better than DE_B_ELM, DE_R_ELM, DE_C_ELM three single-strategy models.
2. Dual-strategy vs multi-strategy
  The experimental settings of the dual-strategy differential evolution algorithm are shown in Table 10. In this group of experiments, except for the different settings of the strategy and the M-SDE, other parameters are consistent with the M-SDE.

**Table 10:** Settings of the dual-strategy models.

Model	Mutation strategy 1	Mutation strategy 2
DE_BC_ELM	DE/best/2	DE/current to best/1
DE_RC_ELM	DE/rand/2	DE/current to best/1
DE_BR_ELM	DE/best/2	DE/rand/2
  Similarly, each model was run independently for ten times, and the performance of each model is shown in Table 11.

**Table 11:** Performance comparison between dual-strategy and multi-strategy of models.

Model	Accuracy (mean ± std)	F1_score (mean ± std)	Precision (mean ± std)
DE_BC_ELM	85.47% ± 0.74%	0.8471 ± 0.0083	0.8564 ± 0.0083
DE_RC_ELM	85.33% ± 0.52%	0.8475 ± 0.0061	0.8575 ± 0.0066
DE_BR_ELM	84.82% ± 0.69%	0.8418 ± 0.0083	0.8519 ± 0.0079
M-SDE_ELM	86.70% ± 0.33%	0.8614 ± 0.0041	0.8702 ± 0.0046
  The accuracy of ten runs of each classifier is shown in Fig. 12.

**Figure 12:**
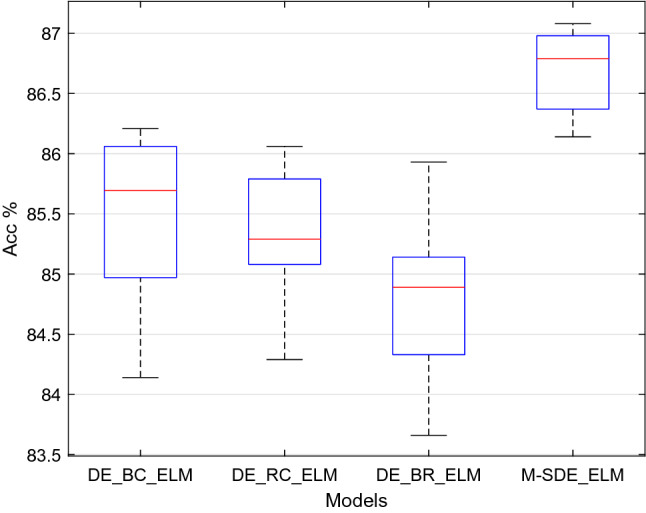
Comparison dual-strategy with multi-strategy models.
  Seen from Table 11, the accuracy of M-SDE_ELM model is 86.70%, F1_score is 0.8614, and precision is 0.8702, respectively 1.23–1.88%, 1.39–1.96%, and 1.27–1.83% higher than the three dual-strategy models. In terms of the standard deviation of the three evaluation indicators, those of the M-SDE_ELM model are the smallest, and combining with Fig. 12, we can see that this model is more stable than other models, and its performance is better.
  Therefore, we can conclude that M-SDE_ELM is better than DE_BC_ELM, DE_RC_ELM, DE_BR_ELM three dual-strategy models. Moreover, we can find that adding the “DE/current to best/1” strategy to the mutation strategy can effectively improve the performance of the model, and combining the three strategies can make the model performance better.
  The above two sets of experimental results show that M-SDE_ELM adopts three mutation strategies, which can effectively improve the performance and stability of the model, and obtains good results.

## Limitations and future scope

This paper proposes the M-SDE method based on the improved DE to optimize the ELM, and builds M-SDE_EnELM so as to carry out the research on birdsongs recognition. The study shows that more research is needed to expand the performance and feasibility of future work, and some of the most important points are listed below:

- The M-SDE method needs to be further optimized to improve the ELM model performance and applicability.
- The categories and sample sizes of birdsongs need to be expanded, and the M-SDE_EnELM model will be extended to more bird audios and other audios recognition.
- Many feature parameter extraction methods are mentioned in^30^. At present, this study only uses MFCC as the feature parameter, and the method proposed in the literature can be tried to extract the features of multiple views for bird audios, and birdsongs recognition can be carried out by combining a variety of different feature parameters.
- Both deep learning and traditional machine learning are now widely used for object recognition^31–33^. Deep learning can also extract low-level features of research objects while classifying. Handcrafted features can retain the characteristics of the research object itself, and combine the features extracted by deep learning with handcrafted features to better express the specific information of the object. Literature^31^ explored two methods of deep learning and machine learning, and combined traditional features and CNN features, and achieved good results. In the future, we will conduct feature fusion with the representation features extracted by deep learning and traditional hand-extracted features to improve the accuracy of of birdsongs recognition.

## Conclusion

This study takes birdsongs as the research object, extracts the feature parameters of differential MFCC, and conducts classification research on birdsongs. The M-SDE method is proposed by improving the standard DE to optimize the ELM model with M-SDE. The ablation experiments show that the use of three strategies in the M-SDE algorithm can effectively improve the performance of the algorithm, so that the optimized ELM model can produce a better classification effect on birdsongs, which is better than the single-strategy models and the dual- strategy models.

Through comparative experiments on the ELM optimized by the three methods of M-SDE, PSO and GOA, the results show that the M-SDE_ELM and ensemble M-SDE_EnELM models proposed in this paper have a classification accuracy of 86.70% and 89.05% in nine species of birds, respectively, which is better than the ELM model optimized by PSO and GOA and the original ELM model. The M-SDE_EnELM model can better solve the problems of unstable performance of a single M-SDE_ELM classifier and difficult to determine the optimal number of neurons in the hidden layer, and has a good generalization ability.

### Ethics declarations

In this paper, the experiments did not use live birds.
